# Supplementary material for: Where diverse populations gather: transit accessibility and the spatial structure of social mixing
Source: Front Big Data. 2026 Jun 18;9:1857064. doi: 10.3389/fdata.2026.1857064 (PMC13322799; doi:10.3389/fdata.2026.1857064)
Supplement: Supplementary file 1 [file Data_Sheet_1.pdf]

Where diverse populations gather: Transit accessibility and the spatial structure of social mixing *Supplementary Material*

1 SUPPLEMENTARY TABLES

Table S1. Spatial clustering of visitor diversity by birth background across Swedish and US cities. Moran's I measures global spatial autocorrelation; HH% and LL% indicate the percentage of POIs in High-High and Low-Low LISA clusters, respectively. All Moran's I values are significant at $p < 0.001$.

Country	City	N POIs	Moran's I	HH%	LL%
Sweden	Gothenburg	16,685	0.237	9.3	14.5
	Helsingborg	5,275	0.161	11.4	10.7
	Linköping	5,311	0.182	7.4	10.1
	Lund	4,824	0.081	5.0	5.7
	Malmö	12,610	0.197	14.3	12.8
	Stockholm	35,842	0.127	6.0	5.6
	Uppsala	7,933	0.167	10.7	10.1
	Västerås	5,263	0.142	9.4	9.7
	Örebro	5,407	0.131	5.3	7.1
US	New York	148,413	0.833	38.6	17.7
	Atlanta	34,354	0.775	26.2	28.9
	Washington DC	24,599	0.667	31.2	16.3

Table S2. Comparison of residential, visitor, and transit catchment diversity by birth background. $^{***}p < 0.001$, $^{**}p < 0.01$, $^{*}p < 0.05$, ^{ns} not significant.

City	N	Res.	Vis.	Cat.	Vis.-Res.	Cat.-Res.	Diff.-in-Diff.
New York	148,413	0.825	0.873	0.887	+0.048***	+0.062***	-0.014***
Washington DC	24,599	0.826	0.840	0.872	+0.014^{ns}	+0.046***	-0.032***
Atlanta	34,354	0.592	0.607	0.626	+0.015***	+0.034***	-0.019***
Gothenburg	16,685	0.575	0.607	0.718	+0.032***	+0.143***	-0.111***
Helsingborg	5,275	0.614	0.598	0.743	-0.015***	+0.130***	-0.145***
Linköping	5,311	0.539	0.530	0.634	-0.008*	+0.095***	-0.103***
Lund	4,824	0.561	0.575	0.645	+0.014***	+0.084***	-0.070***
Malmö	12,610	0.673	0.652	0.806	-0.021***	+0.133***	-0.154***
Stockholm	35,842	0.454	0.583	0.619	+0.129***	+0.166***	-0.037***
Uppsala	7,933	0.539	0.592	0.693	+0.053***	+0.155***	-0.101***
Västerås	5,263	0.601	0.614	0.733	+0.013***	+0.132***	-0.118***
Örebro	5,407	0.480	0.507	0.609	+0.028***	+0.129***	-0.102***

Table S3. GWR summary statistics of spatial heterogeneity in the transit catchment diversity's coefficient across cities. Values report distributional characteristics of local coefficients and model fit. %+ denotes the percentage of locations with positive coefficients; %Hot and %Cold indicate the shares of statistically significant positive and negative coefficients, respectively.

City	Mean	SD	% Positive	%Hot	%Cold	R^2
Stockholm	.187	1.350	53.9	0.5	1.2	.118
Göteborg	.225	1.685	51.1	0.8	0.4	.225
Malmö	-.123	1.731	47.7	0.3	1.1	.182
Uppsala	-.124	2.960	52.5	0.3	2.6	.185
Helsingborg	-.117	1.358	29.3	6.1	8.8	.128
Lund	.081	1.285	55.7	0.1	0.0	.079
Västerås	1.599	8.897	55.9	1.7	1.2	.152
Örebro	-.275	1.729	45.4	1.0	0.0	.141
Linköping	.213	2.090	55.5	1.0	0.1	.186
New York (US)	.150	.834	64.8	32.5	8.1	.794
Atlanta (US)	.118	.317	68.9	12.1	1.5	.766
Washington DC (US)	.915	133.594	62.6	7.5	2.1	.673

Table S4. Logistic regression predicting transit-mixing hotspots. Odds ratios (OR) are reported with 95% confidence intervals. Predictors are standardized (1 unit = 1 SD). $^*p < 0.05$, $^{**}p < 0.01$, $^{***}p < 0.001$. The hotspots are derived from the GWR results for all POIs pooled within each country's cities.

Variable	Sweden (9)	US (3)
Distance to center (km)	.111 [.081, .151]***	1.171 [1.158, 1.185]***
POI density (500 m)	.705 [.646, .769]***	.956 [.945, .967]***
Transit proximity (m)	3.934 [.297, 52.136]	1.301 [1.284, 1.318]***
Pseudo R^2	.026	.023
ROC-AUC	.673	.582
Accuracy	.991	.721
Sensitivity	.000	.044
Specificity	1.000	.983
N (K)	97.8	194.3
Hotspots (%)	.9	27.9
